# Sn(IV)porphyrin-Incorporated TiO_2_ Nanotubes for Visible Light-Active Photocatalysis

**DOI:** 10.3390/molecules29071612

**Published:** 2024-04-03

**Authors:** Nirmal Kumar Shee, Gi-Seon Lee, Hee-Joon Kim

**Affiliations:** Department of Chemistry and Bioscience, Kumoh National Institute of Technology, Gumi 39177, Republic of Korea

**Keywords:** Sn(IV)porphyrin, TiO_2_, nanotubes, photocatalysts, methylene blue dye

## Abstract

In this study, two distinct photocatalysts, namely tin(IV)porphyrin-sensitized titanium dioxide nanotubes (SnP-TNTs) and titanium dioxide nanofibers (TNFs), were synthesized and characterized using various spectroscopic techniques. SnP-TNTs were formed through the hydrothermal reaction of NaOH with TiO_2_ (P-25) nanospheres in the presence of Sn(IV)porphyrin (SnP), resulting in a transformation into Sn(IV)porphyrin-imbedded nanotubes. In contrast, under similar reaction conditions but in the absence of SnP, TiO_2_ (P-25) nanospheres evolved into nanofibers (TNFs). Comparative analysis revealed that SnP-TNTs exhibited a remarkable enhancement in the visible light photodegradation of model pollutants compared to SnP, TiO_2_ (P-25), or TNFs. The superior photodegradation activity of SnP-TNTs was primarily attributed to synergistic effects between TiO_2_ (P-25) and SnP, leading to altered conformational frameworks, increased surface area, enhanced thermo-chemical stability, unique morphology, and outstanding visible light photodegradation of cationic methylene blue dye (MB dye). With a rapid removal rate of 95% within 100 min (rate constant = 0.0277 min^−1^), SnP-TNTs demonstrated excellent dye degradation capacity, high reusability, and low catalyst loading, positioning them as more efficient than conventional catalysts. This report introduces a novel direction for porphyrin-incorporated catalytic systems, holding significance for future applications in environmental remediation.

## 1. Introduction

Semiconductor-based photocatalysis has been widely applied in hydrogen production [1], O_2_ evolution reaction [2], CO_2_ reduction [3], N_2_ fixation [4], wastewater treatment [5], and other fields to tackle the global environmental pollution and energy crisis [6]. Nano-sized frameworks show unique electronic and optical properties that are dependent on the dimensions of photoactive compounds. Given the proven high efficacy of photocatalytic methods, several inorganic- and organic-based micro- or nanomaterials featuring TiO_2_ [7], ZnO [8], zeolites [9], g-C_3_N_4_ [10], bismuth-based photocatalysts [11], fullerenes [12], graphene oxide (GO) or rGO [13], carbon quantum dots [14], and porphyrin-based compounds [15] have been used in semiconductor-based photocatalysis processes. Among them, titanium dioxide (TiO_2_), or titania, has been utilized as a benchmark catalyst due to its low cost, enormous thermo-chemical robustness, nontoxicity, high photodegradation efficiency, and recyclability. However, its high band gap energy (~3.2 eV) prevents the absorption of solar light below the visible light spectrum (λ > 380 nm), hindering the low photocatalytic activity of TiO_2_. The short recombination lifetime of photogenerated reactive pairs in TiO_2_ also limits its photoelectric properties. Furthermore, a substantial quantity of catalysts is required for loading in the photocatalytic reaction to achieve optimal performance [16,17,18]. Therefore, various methods such as photosensitization, metal ion doping, and electro-deposition have been used to extend the solar-harvesting properties of TiO_2_ by enhancing the dissociation of photogenerated active pairs [19,20,21]. Among these methods, photosensitization stands out as a particularly coherent and beneficial technique that has gained widespread use [22,23,24].

Porphyrinoids, including free porphyrin or metalloporphyrins, are proving to be promising photosensitizers due to their high light-harvesting ability in regions of the Soret band (400–470 nm) and Q bands (500–700 nm). Additionally, their inherent aromatic electronic features, substantial flexibility in molecular framework, and rigid structural framework render them notably compelling as photosensitizers, distinguishing them from other optoelectronic materials [25,26,27,28]. In particular, porphyrin-sensitized TiO_2_ hybrid materials have been proven to amplify the interfacial charge transfer, decrease electron–hole recombination rates, and improve photocatalytic performance via heterojunction interfaces [29,30,31,32,33,34].

**Scheme 1:**
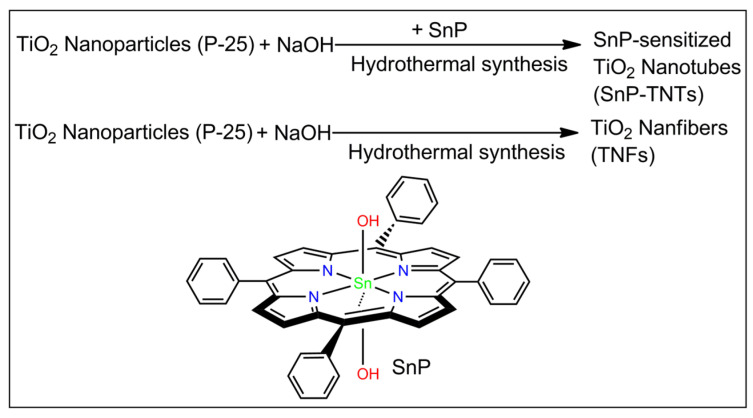
Fabrication of SnP-TNTs and TNFs.

Previously, our group reported the photodegradation of methyl orange dye by Sn(IV) porphyrin-sensitized TiO_2_ nanofibers under the irradiation of visible light. Under basic conditions, a novel heterostructure is produced through a one-step hydrothermal reaction involving *trans*-dihydroxo [5,10,15,20-tetrakis(*p*-tolyl)porphyrinato]Sn(IV) and pure anatase TiO_2_ powder [35]. Sn(IV) porphyrins are ideal building blocks for photo-functional materials used for visible light catalysis. The oxophilic character of the Sn(IV) porphyrin center can easily construct stable six-coordinate diamagnetic complexes with either alkoxides or carboxylates. In particular, the *trans*-dihydroxo complex of the Sn(IV) porphyrin has a strong affinity toward transition metal oxides. These complexes are also known for their unique photo-physical properties. Therefore, structural studies of these compounds via various spectroscopic techniques are of significant value [36,37,38,39,40,41,42]. Within this context, the present study employs a one-step hydrothermal reaction between *trans*-dihydroxo(5,10,15,20-tetraphenylporphyrinato)Sn(IV) (SnP) and TiO_2_ (P-25, 85% anatase and 15% rutile) in basic conditions to fabricate Sn(IV) porphyrin-imbedded TiO_2_ nanotubes (SnP-TNTs) (Scheme 1). To know the impact of Sn(IV) porphyrin on the fabrication of SnP-TNTs, we repeat the above experiments without SnP. Interestingly, we observed TiO_2_ nanofibers (TNFs).

The catalytic property of SnP-TNTs was examined for the solar light-active photodegradation of methylene blue (MB), a highly water-soluble industrial dye. MB is generally utilized as a colorant in the leather, printing, cosmetics, and textile industries, and it is listed as an essential medicine by the World Health Organization. It finds extensive application as a biological stain for managing methemoglobinemia and facilitating endoscopic polypectomy in certain developing nations. Classified as one of the 30 perilous dyes commonly present in wastewater, this substance can induce hypertension, abdominal discomfort, nausea, anemia, and harm in the nervous system. At very low concentrations, MB is carcinogenic, non-biodegradable, and cannot be removed via usual methods like filtration, absorption, precipitation, or coagulation due to its high water solubility [43,44]. Previously, several research groups employed MB dye as a model contaminant to examine the effectiveness of the as-synthesized catalysts [45,46,47,48]. This report outlines various experiments carried out to investigate the optimal conditions associated with the degradation of MB, including analyses of kinetics and the underlying mechanism.

## 2. Results and Discussion

### 2.1. Synthesis and Characterization

The fabrication process of SnP-TNTs is discussed in the experimental section. In a typical procedure, TiO_2_ (P-25), SnP, and NaOH were mixed in water and heated for 24 h in a Teflon-lined stainless-steel autoclave. The adsorbed quantity of SnP was determined to be 0.096 mmol/g by calculating the Sn contents of SnP-TNTs through ICP analysis.

**Figure 1:**
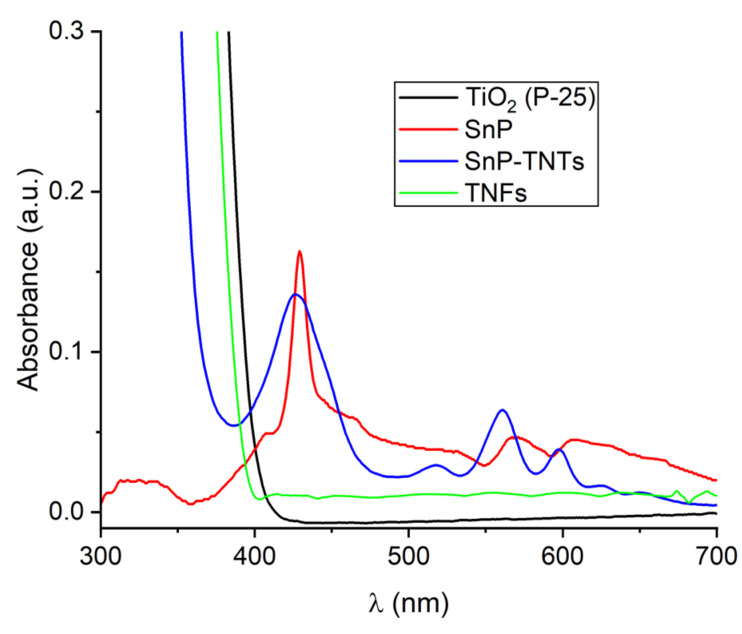
UV-visible spectra of TiO_2_ (P-25), SnP, TNFs, and SnP-TNTs in Nujol.

UV-Vis spectroscopy was utilized to assess the solar-harvesting capabilities of TiO_2_ (P-25), SnP, TNFs, and SnP-TNTs (Figure 1). SnP displayed a broad and strong Soret-band absorption peak at 429 nm, accompanied by two Q-band absorption peaks at 568 and 607 nm. TiO_2_ (P-25) nanoparticles and TNFs showed an intense absorbance peak within the UV spectrum (300–330 nm). Compared to SnP, SnP-TNTs demonstrated a broad and strong Soret-band absorption peak centered at 426 nm, along with three weak Q-band peaks at 517, 560, and 597 nm. Additionally, it exhibited a strong absorbance band within the UV region. These observations suggested a strong binding between tin porphyrin and SnP-TNTs, indicating the formation of a heterostructure rather than a mere physical mixture of SnP and TiO_2_ (P-25). Consequently, SnP-TNTs exhibited broader light absorption across the solar spectrum, enhancing their light-harvesting properties beyond those of either SnP or TiO_2_ (P-25). The band gaps (E_g_), determined via Tauc’s Plot [49] from the absorption data, were approximately 2.37 eV and 3.02 eV for SnP-TNTs, which were smaller than those of TiO_2_ (P-25) (approximately 3.17 eV) or SnP (around 2.79 eV) or TNFs (3.04 eV) (Table S1 and Figure S1). The improved light harvesting and narrower band gap of SnP-TNTs can significantly boost solar energy utilization, generating a larger quantity of photogenerated reactive species vital for catalytic reactions.

The efficiency of separating photogenerated reactive species holds paramount importance in the photodegradation reaction. This aspect was investigated using fluorescence spectroscopy (Figure S2). SnP displayed a fluorescence peak at 654 nm, while SnP-TNTs exhibited two fluorescence peaks at 627 and 648 nm, indicating effective interaction with TiO_2_ (P-25) nanoparticles.

**Figure 2:**
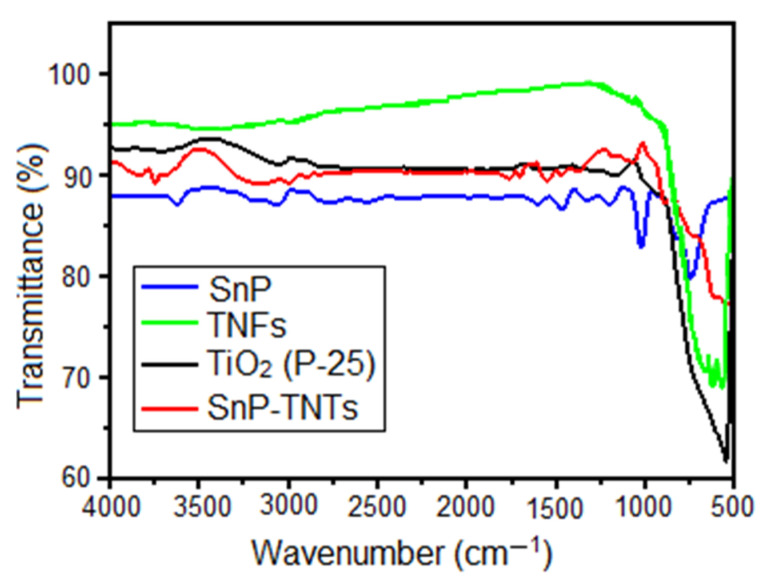
FT-IR spectra of spectra of TiO_2_ (P-25), SnP, TNFs, and SnP-TNTs in KBr.

In the FT-IR spectra of SnP, the peak at 1024 cm^−1^ corresponds to the bending vibration of the aromatic C-H group, while the peak at 793 cm^−1^ is linked to the out-of-plane bending vibration of the aromatic C-H (Figure 2). Additionally, stretching vibrations of C-N and C=C in the pyrrole ring were evident at 1406 cm^−1^ and 1588 cm^−1^, respectively. The stretching vibrations of the axial O-H group were observed at 3594 cm^−1^. Conversely, in TiO_2_ (P-25), prominent absorbance peaks corresponding to Ti–O–Ti stretching were detected between 512 cm^−1^ and 853 cm^−1^. In the case of TNFs, the stretching band of Ti-O-Ti was broadened compared to TiO_2_ (P-25). On the other hand, in the case of SnP-TNTs, the expected stretching band of Ti-O-Ti emerged at 596 cm^−1^. In SnP-TNTs, the characteristic peaks of SnP have been shifted to higher wavenumbers compared to SnP. These observations suggest that the distinctive characteristics of SnP and TiO_2_ (P-25) changed slightly in SnP-TNTs.

**Figure 3:**
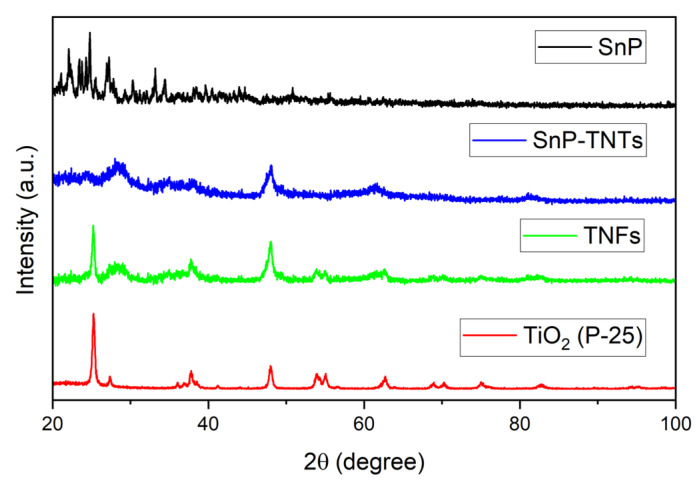
Powder X-ray diffraction patterns of SnP, TiO_2_ (P-25), TNFs, and SnP-TNTs.

The structural patterns of TiO_2_ (P-25), SnP, TNFs, and SnP-TNTs were investigated using an X-ray diffractometer, as shown in Figure 3. SnP displays strong peaks at low angles, ranging from 22 to 34°. TiO_2_ (P-25) exhibited major crystallized peaks centered at 25.2, 37.8, 48.0, 54.0, 55.1, and 62.6°. The commercial TiO_2_ (P-25) nanoparticles used in this study are composed of anatase (85%) and rutile phase (15%). A similar pattern was observed in the case of TNFs, but the peaks are broadened compared to TiO_2_ (P-25). On the other hand, in the case of SnP-TNTs, significant characteristic peaks appeared at 28.2, 47.9, and 61.4°. These observations indicate that all the distinctive features of TiO_2_ (P-25) and SnP were absent in SnP-TNTs. This could be attributed to the low concentration of SnP in SnP-TNTs and drastic changes in the morphologies of TiO_2_ (P-25) nanoparticles.

**Figure 4:**
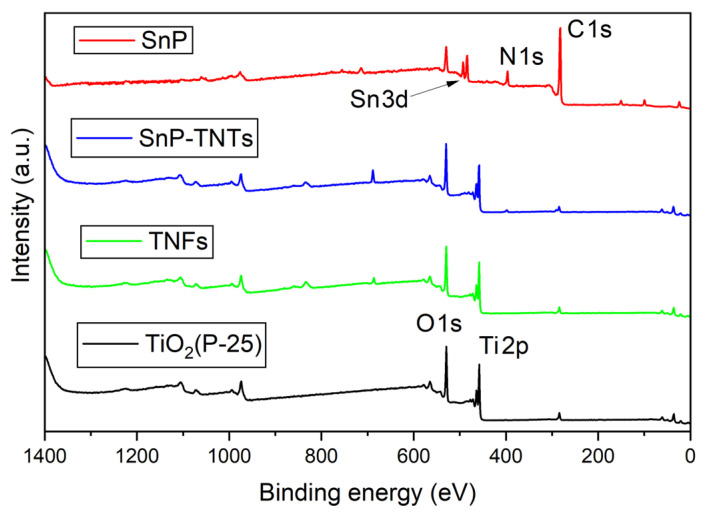
XPS survey spectra of TiO_2_ (P-25), SnP, TNFs, and SnP-TNTs.

In the XPS analysis of TiO_2_ (P-25), SnP, TNFs, and SnP-TNTs, the survey spectra revealed distinct chemical states. According to the survey spectra, SnP consists of Sn, C, N, and O elements; TiO_2_ and TNFs contain Ti and O elements; and SnP-TNTs contain Sn, Ti, C, N, and O elements (Figure 4). In the O 1s spectrum of TiO_2_ (P-25), a singular peak was evident at 529.0 eV (Figure 4). In the case of SnP-TNTs, the O 1s spectrum exhibited a single peak at 529.7 eV. On the other hand, the O 1s spectrum of TNFs showed a single peak at 529.5 eV. Regarding the Ti 2p spectrum, TiO_2_ (P-25) displayed two peaks at 463.3 eV (Ti 2p_1/2_) and 457.5 eV (Ti 2p_3/2_). However, in SnP-TNTs, these peaks shifted to 464.2 eV and 458.4 eV, indicating a higher binding energy. This suggests a reduced Ti 2p electron density, likely due to strong interactions between the hydroxyl group in SnP and the Ti atoms on the TiO_2_ (P-25) surface. This higher binding energy potentially signifies interfacial electron transfer between them. On the other hand, the Ti 2p spectrum of TNFs exhibited peaks at 463.9 eV (Ti 2p_1/2_) and 458.1 eV (Ti 2p_3/2_). Deconvoluted profiles for O 1s, Sn 3d, C 1s, and Ti 2p are displayed in Figure S3, although the relative intensities of N 1s, C 1s, and Sn 3d were too low for proper analysis. SnP showed two peaks at 493.0 (Sn 3d_3/2_) and 484.5 (Sn 3d_5/2_) eV for the Sn 3d core. On the other hand, the peak positions of these two peaks were slightly changed in the case of SnP-TNTs (492.8 eV for Sn 3d_3/2_ and 484.3 eV for Sn 3d_5/2_) compared to SnP. In the case of the C 1s core spectrum, SnP exhibited three peaks at 284.1 eV, 282.5 eV, and 281.8 eV. On the other hand, these peaks were changed in the case of SnP-TNTs (285.5 eV, 284.2 eV, and 283.7 eV). This observation implies that the pi-electron density of porphyrin transferred to the hybrid core systems.

The TGA curves of all samples are depicted in Figure S4. SnP shows a moderate weight loss (~5%) between 50 °C and 320 °C due to absorbed water, followed by the disintegration of the aromatic ring from 350 to 700 °C. TiO_2_ (P-25) only manifests a 3% weight loss between 50 and 900 °C. In the case of SnP-TNTs, TGA analysis confirmed a 14.9% weight loss between 200 and 450 °C. This weight loss evidently supports the incorporation of SnP into SnP-TNTs. On the other hand, 8.8% weight loss was observed for TNFs.

To determine the permanent porosities of SnP-TNTs and TNFs, the BET surface area was measured using N_2_ adsorption–desorption isotherms. SnP-TNTs possessed a moderate specific surface area of 152 m^2^ g^−1^ (Figure S5), with an estimated pore volume of 0.47 cm^3^ g^−1^. SnP-TNTs exhibited a type-IV adsorption–desorption isotherm, suggesting a mesoporous nature. On the other hand, TNFs possessed a low specific surface area of 112 m^2^ g^−1^, with an estimated pore volume of 0.31 cm^3^ g^−1^. TNFs showed a type-II adsorption–desorption isotherm. In contrast, TiO_2_ (P-25) exhibited a much lower surface area of only 51.6 m^2^ g^−1^ [50]. Therefore, the presence of a base facilitated the strong coupling of TiO_2_ (P-25) nanoparticles with SnP under hydrothermal conditions, resulting in the fabrication of thermally stable mesoporous materials, SnP-TNTs. The higher BET surface area of SnP-TNTs is probably due to its amorphous nature compared to TiO_2_ (P-25).

**Figure 5:**
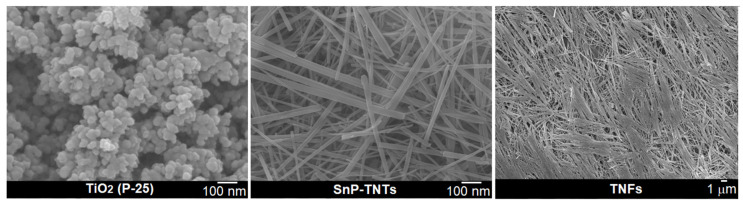
FE-SEM images for TiO_2_ (P-25), SnP-TNTs, and TNFs.

FE-SEM techniques were employed to assess the morphologies of SnP-TNTs in comparison to TiO_2_ (P-25). In the FE-SEM image of TiO_2_ (P-25), spherical nanospheres were evident, exhibiting an average diameter ranging from 50 to 70 nm (Figure 5). SnP-TNTs revealed consistent nanotubes characterized by an average length spanning 300 to 500 nm and a width varying from 10 to 20 nm. Interestingly, when pure anatase TiO_2_ was used previously, the observation was SnP-intercalated trititanate nanofibers. These nanofibers have lengths in the range of 0.5–1 μm with an average diameter of approximately 50 mm [35]. However, when TiO_2_ (P-25, 85% anatase, and 25% rutile) was used in place of pure anatase TiO_2_, nanotubes formed. On the other hand, in the absence of SnP, TiO_2_ (P-25) nanospheres transformed into nanofibers (TNFs) under identical conditions. The lengths of these nanofibers vary from a few micrometers to millimeters. Notably, SnP did not exhibit any nano-scale morphology [34].

**Figure 6:**
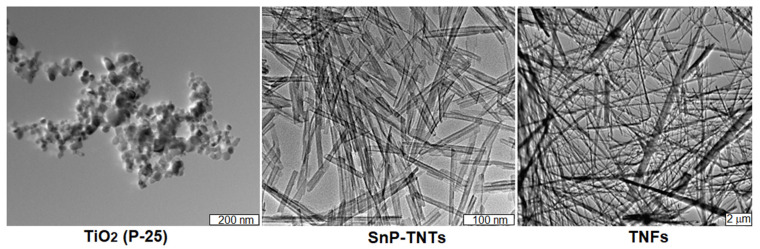
TEM images for TiO_2_ (P-25), SnP-TNTs, and TNFs.

The TEM technique was employed to further investigate the morphologies of SnP-TNTs in comparison to TiO_2_ (P-25). Similar to the FE-SEM results, TiO_2_ (P-25) displayed nanospheres, while SnP-TNTs showcased nanotubes with hollow spaces. In the case of TNFs, the observation included nanofibers along with a small percentage of nanotubes (Figure 6). Enlarged FE-SEM and TEM of nanotubes for SnP-TNTs are shown in Figure S6. Energy dispersive X-ray spectroscopy (EDS) mapping images (Figure S7) show that SnP-TNTs preserve the nanotube skeleton, in which O, N, C, Ti, and Sn elements are homogeneously distributed.

### 2.2. Photocatalytic Degradation of MB Dye

**Figure 7:**
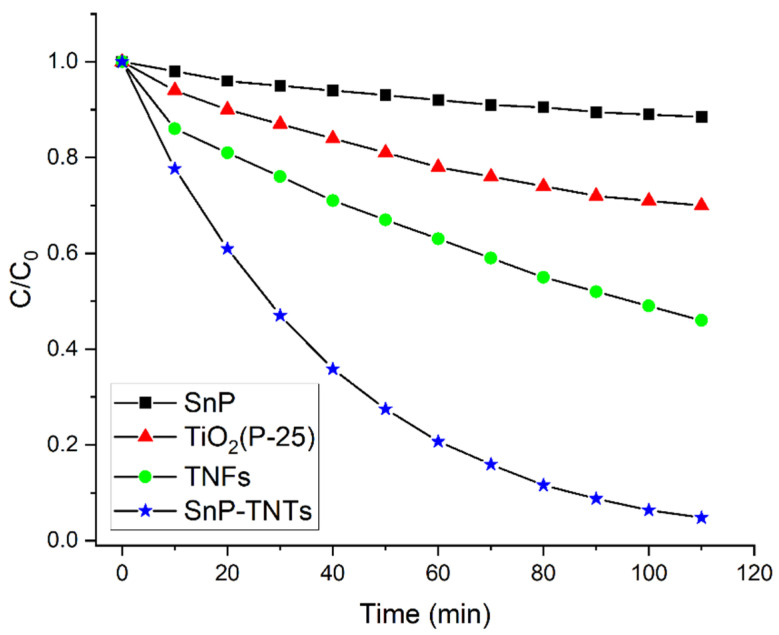
Photocatalytic degradation of MB dye by SnP-TNTs, TNFs, SnP, and TiO_2_ (P-25) under visible light irradiation (pH 7, 298 K).

The catalytic efficiencies of SnP, TiO_2_ (P-25), SnP-TNTs, and TNFs were evaluated through the visible light-induced degradation of MB dye. Figure S8 highlights the requirement of approximately 40 min to achieve the adsorption–desorption equilibrium. SnP, TiO_2_ (P-25), TNFs, and SnP-TNTs displayed physical adsorption levels of about 4.3%, 15%, 20%, and 25% of MB dye, respectively. This confirmed the mesoporous frameworks of TiO_2_ nanostructures, enhancing the absorptivity of SnP-TNTs and TNFs while facilitating mass diffusion. The visible light-induced time-bound absorption spectra of MB in the presence of SnP-TNTs are illustrated in Figure S9. Figure S9 reveals a reduction in MB absorbance at 660 nm with an increasing visible light irradiation time. Additionally, Figure 7 depicts significant progress in MB decay across all catalysts.

The degradation coefficient, expressed as (*C*_0_ − *C*)/*C*_0_, was utilized to assess the photodegradation of MB, where *C*_0_ and *C* represent the MB concentrations at time zero and time *t*, respectively. The calculated photodegradation rates for MB were 13% for SnP, 30% for TiO_2_ (P-25), 54% for TNFs, and 95% for SnP-TNTs over 110 min of visible-light exposure (Figure 7). Notably, SnP-TNTs demonstrated the highest efficiency in removing MB during photodegradation compared to SnP, TiO_2_ (P-25), and TNFs.

To delve deeper into the reaction kinetics of MB photodegradation, the pseudo-first-order theory was applied, using the equation ln(*C*_0_/*C*) = *kt*. This model is commonly employed for catalytic photodegradation reactions involving low initial dye concentrations, where *k* represents the photodegradation rate constant. Figure S10 illustrates the reaction kinetics of MB photodegradation based on the data from Figure 7. The photodegradation rate constants for MB were determined to be 0.0011 min^−1^ for SnP, 0.0034 min^−1^ for TiO_2_ (P-25), 0.0067 min^−1^ for TNFs, and, notably, 0.0277 min^−1^ for SnP-TNTs (Figure S10). These degradation rate constants were noteworthy when compared to previously published values for MB photodegradation (Table S2). Specifically, the photodegradation rate constant of MB by SnP-TNTs (0.0277 min^−1^) significantly surpassed those of anatase TiO_2_ (0.0090 min^−1^) [45] and TiO_2_ NPs (0.0180 min^−1^) [46]. Moreover, it remained comparable to the photodegradation rates exhibited by porphyrin-based nanostructures (0.0280 min^−1^) [47] or porphyrin-based metal–organic frameworks (0.0200 min^−1^) [48].

Among all the catalysts, SnP-TNTs exhibited the highest degradation rate constant within 110 min, capable of degrading 95% of the MB dye. To investigate the role of SnP in SnP-TNTs’ catalytic efficiency, various SnP-TNTs were prepared with differing wt% of SnP for TiO_2_ (P-25), and their MB dye removal efficiencies were measured (Figure S11). The photodegradation efficiencies of SnP-TNTs (X = 0.1 mmol) surpassed those of TiO_2_ (P-25) or SnP individually. As the molar mass of SnP increased in the hybrid compared to TiO_2_ (P-25), the degradation rate rose, reaching its peak at 0.1 mmol. Following this peak, the rates exhibited a slight decrease to around 85 or 80%. These observations suggest the existence of synergistic effects between TiO_2_ (P-25) and SnP, contributing significantly to the exceptional photodegradation activity observed in SnP-TNTs.

The recovery process for SnP-TNTs from the reaction vessel proved notably simpler compared to both SnP and TiO_2_ (P-25). Initially, solid materials were filtered post-experiment, followed by rinsing with H_2_O and subsequent drying at 70 °C for 4 h in an oven. Assessing a catalyst’s reusability is crucial for industrial applications. Consequently, recycling tests were conducted for SnP-TNTs for the photodegradation of MB dye (Figure S12). After 10 cycles, SnP-TNTs maintained high degradation efficiency toward MB dye photodegradation, with an only 5% decrease. This outcome indicates the remarkable stability of SnP-TNTs as a photocatalyst. Furthermore, to corroborate the stability of this compound, the morphology of SnP-TNTs post-photodegradation experiments were conducted. TEM images (Figure S13) of SnP-TNTs used for degradation studies before and after exhibited striking similarity, indicating an unchanged mesoporous network during the photodegradation process.

The optimization of reaction conditions for MB dye degradation included investigating the dye-to-catalyst ratio, pH of the MB solution, and reaction temperature. Multiple photodegradation experiments were conducted at varying temperatures to observe the temperature-dependent photodegradation of MB by SnP-TNTs. Remarkably, the photodegradation performance demonstrated an increase with rising temperatures (Figure S14). Initially, MB solutions were prepared in distilled water with a pH of 7.0. However, it was observed that the pH of the MB solution significantly influenced the degradation efficiency of MB dye (Figure S15). The photodegradation rate exhibited an increase with increasing pH from 2 up to 8, followed by a decrease beyond pH 8 up to 12. The effect of a basic medium (high pH) on photodegradation was less pronounced compared to an acidic medium (low pH). Exploring the dependence of the dye-to-catalyst ratio on the photodegradation rate of MB, different MB solutions with concentrations ranging from 5 to 50 mg/L were prepared, using a consistent quantity of SnP-TNTs (50 mg each time). Interestingly, it was observed that the photodegradation rate decreased with an increasing MB concentration (Figure S16).

The potential mechanism behind the catalytic photodegradation reaction by SnP-TNTs differs from that of TNFs. In the case of TNFs, the degradation mechanism consists of five steps, as summarized in Equations (1)–(5). It begins with the catalyst TiO_2_ absorbing light upon irradiation. Valence band (VB) electrons are promoted to the CB band upon crossing the band gap, resulting in the generation of photogenerated pairs (h^+^/e^−^) on the catalyst’s surface. Subsequently, photogenerated holes (h^+^) react with water to form ^•^OH. The excited electrons, in turn, may react with O_2_ to produce O_2_^−•^. These highly reactive species (O_2_^−•^ and ^•^OH) are instrumental in decomposing the MB dye into smaller fragments, ultimately resulting in low-toxic CO_2_ and H_2_O. In summary, the proposed mechanism for a catalyst (cat) is

cat + *h*ν → cat * (e^−^ + h^+^) (1)

h^+^ + H_2_O → ^•^OH + H^+^ (2)

O_2_ + e^−^ → O_2_^−•^ (3)

O_2_^−•^ + MB → degraded products (4)

^•^OH + MB → degraded products (5)

On the other hand, in the case of SnP-TNTs, both the TiO_2_ nanostructure and SnP can be concurrently excited upon light absorption, generating hole–electron pairs (*h*^+^/*e*^−^). Consequently, a charge transfer mechanism is also possible in this case. The electrons from SnP smoothly transfer to the conduction band (CB) of TiO_2_ through the interface between Ti atoms in TiO_2_ and the hydroxyl group in SnP. These excited electrons may further react with O_2_ to produce superoxide radical anions (O_2_^−•^), contributing to the degradation of MB dye. Additionally, the photogenerated holes in TiO_2_ may react with H_2_O to create hydroxyl radicals (^•^OH), which also aid MB degradation [51,52,53].

SnP + *h*ν → SnP* (6)

SnP* + MB → SnP^−•^ + MB^+^ (oxidized product) (7)

SnP^−•^ +TiO_2_ → SnP + TiO_2_ (e^−^) (8)

TiO_2_ (e^−^) + MB → TiO_2_ + MB^−^ (reduced product) (9)

SnP-TNTs exhibit higher light absorption capacity compared to TNFs. The presence of SnP increased the recombination time by stabilizing the photogenerated active pairs over the surface of conjugated porphyrin networks. Moreover, the incorporation of SnP-forming TNTs creates a large surface area and higher active sites for cationic dye degradation compared to TNFs. The integration of porphyrin likely not only enhanced the surface properties of TiO_2_ but also facilitated electron transport from the CB of SnP to the CB of TiO_2_ [54] compared to TNFs or TiO_2_ (P-25).

To identify the photogenerated active species during the degradation process by SnP-TNTs, radical trapping tests were conducted [55,56,57,58]. *tert*-Butanol (*^t^*BuOH) was used to detect ^•^OH, *para*-benzoquinone (*p*-BQ) for O_2_^−•^, ethylenediaminetetraacetic acid disodium (Na_2_-EDTA) for *h*^+^, and sodium azide (NaN_3_) for ^1^O_2_ (Figure S17). The analysis confirmed that the presence of Na_2_-EDTA, *p*-BQ, and *^t^*BuOH significantly impacted degradation efficiency. Photogenerated holes (*h*^+^) emerged as the primary reactive components responsible for photodegradation, overshadowing the contributions of O_2_^−•^ or ^•^OH. Notably, the presence of NaN_3_ had no effect on the decomposition of MB. In the absence of either light or catalyst (Figure S17), minor decay of MB was observed, emphasizing the necessity of both light and catalyst for MB photodegradation.

Exploring the degradation of MB under various monochromatic light wavelengths (Figure S18) revealed the impact of light intensity on SnP-TNTs’ performance. Optical absorption was identified as a significant contributor to solar energy conversion and photocatalytic efficiency. Additionally, SnP-TNTs displayed heightened degradation efficiency at the junction of UV and visible light regions (400 to 500 nm).

Electrospray ionization mass spectrometry (ESI-MS) was utilized to examine the features of the degradation products of MB dye. An analysis of samples taken after 60 min during the photodegradation reaction (Figure S19) revealed new peaks, indicating the photodegradation of MB into low-molecular-weight fragments [59,60]. Potential intermediates for the photodegradation of MB were depicted based on the ESI-MS results (Figure S20). Initially, the base peak (*m*/*z* = 284.1; [MB–Cl]^+^) corresponded to the MB dye. Subsequent N-de-ethylation led to the formation of chromophoric species (*m*/*z* 228.0), followed by ring cleavage, resulting in fragments with molecular weights of *m*/*z* 119.0 and 111.0. An oxidative ring opening product (*m*/*z* 304.1) formed from MB, which further fragmented into molecular weights of *m*/*z* 175.0 and *m*/*z* 95.0. These lower-molecular-weight intermediates underwent aromatic ring breaks and hydrolysis, ultimately yielding a compound with *m*/*z* 119.0. Ultimately, these low-molecular-weight intermediates were mineralized into nontoxic CO_2_ and H_2_O. Additionally, the total organic carbon (TOC) removal percentage was calculated to evaluate MB photodegradation [61], indicating that SnP-TNTs achieved a TOC removal value of 84% for MB photodegradation.

## 3. Materials and Methods

TiO_2_ (Degussa, P-25, crystalline composition: anatase (85%) and rutile (15%)) was procured from Sigma-Aldrich (St. Louis, MO, USA). Other chemicals were obtained from TCI. *trans*-Dihydroxo-(5,10,15,20-tetraphenylporphyrinato) Sn(IV) (SnP) was synthesized based on a previously published method [62]. Steady-state UV-vis spectra were recorded using a Shimadzu UV-3600 spectrophotometer (Shimadzu, Tokyo, Japan) in Nujol. Fluorescence spectra were recorded with a Shimadzu RF-5301PC fluorescence spectrophotometer (Shimadzu, Tokyo, Japan) in Nujol. Fourier transform infrared spectroscopy (FT-IR) spectra (KBr method) were obtained using a Shimadzu FTIR-8400S spectrophotometer (Shimadzu, Tokyo, Japan). Thermogravimetric analysis (TGA) was performed using an Auto-TGA Q500 instrument (TA Instruments, New Castle, PA, USA) at a scan rate of 10 °C/min, from 30 to 900 °C, and under an N_2_ atmosphere. The Brunauer-Emmett-Teller (BET) surface area was determined using an analyzer (BELSORP-mini volumetric adsorption equipment) using N_2_ adsorption isotherms at 77 K. The data on surface and porous size were obtained by using Autosorb-iQ and Quadrasorb SI. Powder X-ray diffraction (PXRD) patterns were obtained using a Bruker AXS D8 Advance powder X-ray diffractometer (Bruker, Billerica, MA, USA). The morphology and elemental distribution of the synthesized samples were investigated using a field emission scanning electron microscope (FE-SEM) (MAIA III, TESCAN, Brno, Czech Republic). Transmission electron microscope (TEM) images were obtained by using JEOL/JEM 2100 with energy dispersive X-ray spectroscopy (EDS). An X-ray photoelectron spectroscopy (XPS) instrument (Thermo Fisher Scientific, Waltham, MA, USA) equipped with a micro-focused Al Kα source was used to analyze the elemental composition of the sample. All the samples were deposited on the surface of copper tape via the drop-casting method after stirring in water. The inductively coupled plasma (ICP) analyses were performed on an ICP-Spectrociros CCD instrument. For visible light sources, we used a 150 W xenon arc lamp (ABET technologies, Old Gate Lane Milford, CT, USA).

### 3.1. Synthesis of Sn(IV)porphyrin-Imbedded TiO_2_ Nanotubes (SnP-TNTs)

SnP-TNTs were fabricated via a straightforward one-step hydrothermal method. About 77 mg of SnP (0.1 mmol) was added to ethanol (20 mL), followed by continuous stirring. After that, an 80 mL aqueous solution of sodium hydroxide (5 M) was added through continuous stirring. Then, 1 g of TiO_2_ (P-25) powder (0.013 mol) was mixed into the solution. After that, the heterogeneous mixture was transferred into a 150 mL Teflon-lined stainless-steel autoclave. The hydrothermal process was maintained at 200 °C in an atmospheric environment for 24 h. After, deionized water (500 mL) was added, and the mixture was then centrifuged. The precipitate was filtered through a 0.1 µm VCTP-membrane Millipore filter and rinsed consecutively with water (200 mL) and ethanol (200 mL) to extract physically absorbed SnP from the surface of precipitates. Solid SnP-TNTs were acquired after drying at 70 °C for 6 h (yield: 0.940 g).

### 3.2. Synthesis of TiO_2_ Nanofibers (SnP-TNFs)

For the fabrication of SnP-TNFs, we followed a similar procedure of SnP-TNTs except for the addition of SnP. Solid SnP-TNFs were prepared after drying at 70 °C for 6 h (yield: 0.910 g).

### 3.3. Photocatalytic Degradation Reaction

The catalytic performance of SnP-TNTs was examined through the decay of MB dye under the irradiation of a visible light source (a 150 W xenon arc lamp; ABET Technologies, Old Gate Lane Milford, CT, USA) with a UV cut-off filter at room temperature (298 K). Then, 50 mg of SnP-TNTs was mixed with 200 mL of MB solution (20 mg L^−1^ in deionized H_2_O, pH 7.0) while stirring. The adsorption–desorption equilibrium was reached after keeping the heterogeneous mixture in the dark for 40 min. After exposure to visible light, 4 mL of the reaction mixture was transferred for 10 min into a test tube. SnP-TNTs were removed from the reaction solution via centrifugation, followed by filtration. After that, a UV-Vis spectrophotometer was used to measure the residual concentration of MB by observing the absorbance at 660 nm.

## 4. Conclusions

Two photocatalysts SnP-TNTs and TNFs were fabricated through the hydrothermal reaction of NaOH with TiO_2_ (P-25) in the presence or absence of SnP, respectively. Generally, TiO_2_ (P-25) appeared as a spherical nanospheres with an average diameter ranging from 50 to 70 nm. The morphologies of SnP-TNTs appeared as regular nanotubes with an average length varying from 300 to 500 nm and a width ranging from 10 to 20 nm. On the other hand, TNFs appeared as nanofibers. Compared to SnP, TiO_2_ (P-25), or TNFs, SnP-TNTs dramatically improved visible light photodegradation of contaminants via the synergistic effects between TiO_2_ and SnP. Incorporating SnP onto the surface of TiO_2_ (P-25) not only controlled the conformational frameworks but also created high structural modifications that induced high thermo-chemical stability, high surface area, unique morphology, and remarkable visible light photodegradation performance against cationic MB dye. SnP-TNTs removed almost 95% MB dye within 100 min (rate constant = 0.0277 min^−1^). The excellent dye photodegradation capacity, high reusability, and low catalyst loading proved the superior efficiency of SnP-TNTs compared to conventional catalysts. This study showcases a new direction for the development of porphyrin-incorporated catalytic systems and holds great significance for extending their future applications in water remediation.

## Data Availability

Data are contained within the article and Supplementary Materials.

## Supplementary Materials

The following supporting information can be downloaded via this link: https://www.mdpi.com/article/10.3390/molecules29071612/s1. Table S1. Band gap energy (E_g_) calculated from the plots of (αhν)^2^ versus (hν) using Tauc’s Plot method. Table S2. Photodegradation efficiency of MB dye using different photocatalysts. Figure S1. Determination of band gap energy of SnP, SnP-TNTs, TNFs, and TiO_2_. Figure S2. Fluorescence spectra of SnP, and SnP-TNTs in Nujol (λ_ex_ = 550 nm). Figure S3. XPS spectra of SnP-TNTs and TiO_2_ (P-25). Deconvoluted profiles of the O 1s, Ti 2p, C 1s, and Sn 3d core levels. Figure S4. TGA curves of SnP, TiO_2_ (P-25), TNFs, and SnP-TNTs. Figure S5. N_2_ adsorption–desorption isotherms of SnP-TNTs and TNFs (right); corresponding pore size distribution curve (left). Figure S6. Enlarged FE-SEM and TEM images for SnP-TNTs. Figure S7. Energy dispersive X-ray spectroscopy (EDS) elemental maps (C, N, O, Ti, and Sn) of SnP-TNTs derived from TEM analysis. Figure S8. Time-dependent adsorption ratios for MB dye using SnP, TiO_2_ (P-25), SnP-TNTs, and TNFs. Figure S9. Visible light-driven photocatalytic degradation of MB dye in aqueous solution using SnP-TNTs. Figure S10. Comparative kinetics of photocatalytic degradation of MB dye under visible light irradiation using SnP, TiO_2_ (P-25), SnP-TNTs, and TNFs as photocatalysts. Figure S11. Comparison of MB dye degradation in the presence of different SnP-TNTs (SnP concentration relative to TiO_2_ P-25). Figure S12. Cyclability of the composite photocatalyst SnP-TNTs in the degradation of MB dye. Figure S13. TEM of composite photocatalyst SnP-TNTs, before and after the MB dye degradation experiment. Figure S14. Temperature-dependent degradation of MB Dye in the presence of composite photocatalyst SnP-TNTs. Figure S15. Effect of pH on the degradation of MB dye in the presence of composite photocatalyst SnP-TNTs. Figure S16. Effect of initial concentration of MB dye on degradation experiments using 50 mg of composite photocatalyst SnP-TNTs. Figure S17. Photocatalytic degradation of MB dye in aqueous solution by the composite photocatalyst SnP-TNTs with different scavengers under visible light irradiation ([Na_2_-EDTA]_0_ = [p-BQ]_0_ = [NaN_3_]_0_ = [^t^BuOH]_0_ = 1 mM, pH 7.0, T = 298 K). Figure S18. Photocatalytic activities of SnP-TNTs at different wavelengths for the degradation of MB dye. Figure S19. ESI-mass spectrum (positive-ion mode) of the reaction mixture of MB dye with the composite photocatalyst SnP-TNTs after 60 min of visible light irradiation. Figure S20. Possible intermediates in the degradation pathway of MB dye in the presence of composite photocatalyst SnP-TNTs. References [45,46,47,48,63,64,65,66,67,68,69,70,71,72,73,74,75,76,77,78,79,80,81,82,83,84,85,86,87,88,89,90,91,92,93] are cited in the text.
