# Supplementary material for: Sn(IV)porphyrin-Incorporated TiO2 Nanotubes for Visible Light-Active Photocatalysis
Source: Molecules. 2024 Apr 3;29(7):1612. doi: 10.3390/molecules29071612 (PMC11013583; doi:10.3390/molecules29071612)
Supplement: Supplementary file 1 [file molecules-29-01612-s001.zip › molecules-2903438-supplementary.pdf]

Supplementary Materials

Sn(IV) Porphyrin-Incorporated TiO_2 Nanotubes for Visible Light-Active Photocatalysis

Nirmal Kumar Shee, Gi-Seon Lee, and Hee-Joon Kim*

Department of Chemistry and Bioscience, Kumoh National Institute of Technology, Gumi 39177, Republic of Korea

List of contents:

Table S1. Band gap energy (E_g) calculated from the plots of $(\alpha h\nu)^2$ versus $(h\nu)$, using the Tauc's Plot method.

Table S2. Photodegradation efficiency of MB dye using different photocatalysts.

Figure S1. Determination of band gap energy of SnP, SnP-TNTs, TNFs, and TiO_2.

Figure S2. Fluorescence spectra of SnP, and SnP-TNTs in Nujol (λ_{ex} = 550 nm).

Figure S3. XPS spectra of SnP-TNTs and TiO_2 (P-25). Deconvoluted profiles of the O 1s, Ti 2p, C 1s, and Sn 3d core levels.

Figure S4. TGA curves of SnP, TiO_2 (P-25), TNFs, and SnP-TNTs.

Figure S5. N_2 adsorption-desorption isotherms of SnP-TNTs and TNFs (right); corresponding pore size distribution curve (left).

Figure S6. Enlarged FE-SEM and TEM images for SnP-TNTs

Figure S7. Energy dispersive X-ray spectroscopy (EDS) elemental maps (C, N, O, Ti, and Sn) of SnP-TNTs derived from TEM analysis.

Figure S8. Time-dependent adsorption ratios for MB dye using SnP, TiO_2 (P-25), SnP-TNTs, and TNFs.

Figure S9. Visible light-driven photocatalytic degradation of MB dye in aqueous solution using SnP-TNTs.

Figure S10. Comparative kinetics of photocatalytic degradation of MB dye under visible light irradiation using SnP, TiO_2 (P-25), SnP-TNTs, and TNFs as photocatalysts.

Figure S11. Comparison of MB dye degradation in the presence of different SnP-TNTs (SnP concentration relative to TiO_2 P-25).

Figure S12. Cyclability of the composite photocatalyst SnP-TNTs in the degradation of MB dye.

Figure S13. TEM of composite photocatalyst SnP-TNTs, before and after the MB dye degradation experiment.

Figure S14. Temperature-dependent degradation of MB Dye in the presence of composite photocatalyst SnP-TNTs.

Figure S15. Effect of pH on the degradation of MB dye in the presence of composite photocatalyst SnP-TNTs.

Figure S16. Effect of initial concentration of MB dye on degradation experiments using 50 mg of composite photocatalyst SnP-TNTs.

Figure S17. Photocatalytic degradation of MB dye in aqueous solution by the composite photocatalyst SnP-TNTs with different scavengers under visible light irradiation ($[Na_2\text{-}EDTA]_0 = [p\text{-}BQ]_0 = [NaN_3]_0 = [^tBuOH]_0$ = 1mM, pH 7.0, T = 298 K).

Figure S18. Photocatalytic activities of SnP-TNTs at different wavelengths for the degradation of MB dye.

Figure S19. ESI-MS spectrum (positive ion mode) of the reaction mixture of MB dye with the composite photocatalyst SnP-TNTs after 60 min of visible light irradiation.

Figure S20. Possible intermediates in the degradation pathway of MB dye in the presence of composite photocatalyst SnP-TNTs.

Table S1. Band gap energy (E_g) calculated from the plots of $(\alpha h v)^2$ versus (hv), using the Tauc's Plot method.

Sample	Band gap energy (eV)
TiO_2 (P-25)	3.17
SnP	2.79
SnP-TiO_2 nanotubes (SnP-TNTs)	2.37 and 3.02
TNFs	3.04

Table S2. Photodegradation efficiency of MB dye using different photocatalysts.

Photocatalysts	Rate constant (min^{-1})	References
Polyaniline	0.0181	[1]
Polyaniline/ZnO	0.0241	[1]
CdO	0.0051	[2]
CdO/PANI	0.0197	[2]
$Fe_{0.01}Ni_{0.01}Zn_{0.98}O$	0.0204	[3]
$Fe_{0.01}Ni_{0.01}Zn_{0.98}O$/PANI	0.0224	[3]
TiO_2-Fe_3O_4-Bentonite	0.0324	[4]
TiO_2/rGO	0.0045	[5]
TiO_2/rGO/Ag	0.0170	[5]
Nano-Metallic Particles	0.0390	[6]
P2ABSA/TiO_2	0.0138	[7]
TiO_2/ poly-o-phenylenediamine	0.0098	[8]
Anatase TiO_2	0.0090	[9]
TiO_2/$GO_{0.4}$	0.0520	[9]
TiO_2 NPs	0.0180	[10]
ZnO/$NiFe_2O_4$	0.0289	[11]
CdS	0.0120	[12]
Bi_2WO_6	0.0150	[12]
Bi_2WO_6–CdS	0.0220	[12]
PVA-assisted Bi_2WO_6–CdS	0.0240	[12]
$Li_{0.9}Ta_{0.8}W_{0.25}O_3$	0.0401	[13]
Fe_2O_3/graphene/CuO	0.0725	[14]
PVDF/GO/ZnO	0.0220	[15]
MoS_2/TiO_2	0.0190	[16]
Pristine CeO_2	0.0121	[17]
rGO(1.0 wt%)–TiO_2	0.0762	[18]
Fe_2TiO_5	0.0160	[19]
$MnTiO_3$	0.0052	[20]
Nitrogen doped $SrTiO_3$	0.0149	[21]
Chitosan bi-metal oxide	0.0696	[22]
$Eu_{0.5}$/Bi_2SiO_5	0.0107	[23]
CuO/GO	0.0741	[24]
ZnO Nanorods	0.0060	[25]
ZIF-8	0.0056	[26]
ZIF-8/NDCQDs	0.0103	[26]
Peroxymonosulfate/Mn_3O_4	0.0050	[27]
Au-TiO_2	0.0067	[28]
p-$CuFe_2O_4$	0.0071	[29]
n-SnO_2/*p*-$CuFe_2O_4$	0.0167	[29]
Bi-Fe/TiO_2	0.0120	[30]
CS-TiO_2 NPs	0.0398	[31]
Fe-Co-SiO_2/H_2O_2	0.0630	[32]
CuO/CuS/MnO_2	0.0220	[33]
ZnP-SnP-ZnP triad nanostructure	0.0280	[34]
SnP-Ag MOF	0.0200	[35]
SnP	0.0011	this study
TiO_2 (P-25)	0.0034	this study
TNFs	0.0067	this study
SnP-TNTs	0.0277	this study

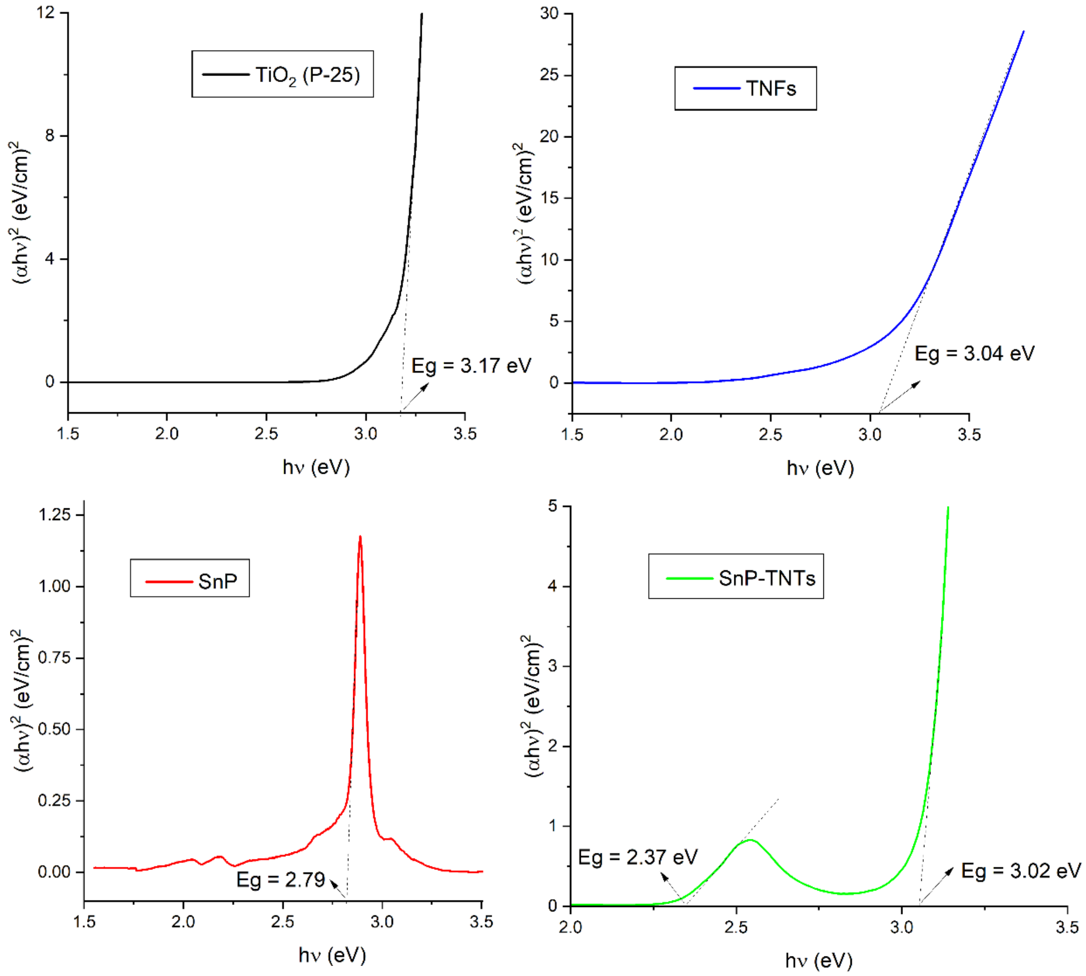

Figure S1. Determination of band gap energy of SnP, SnP-TNTs, TNFs, and TiO_2.

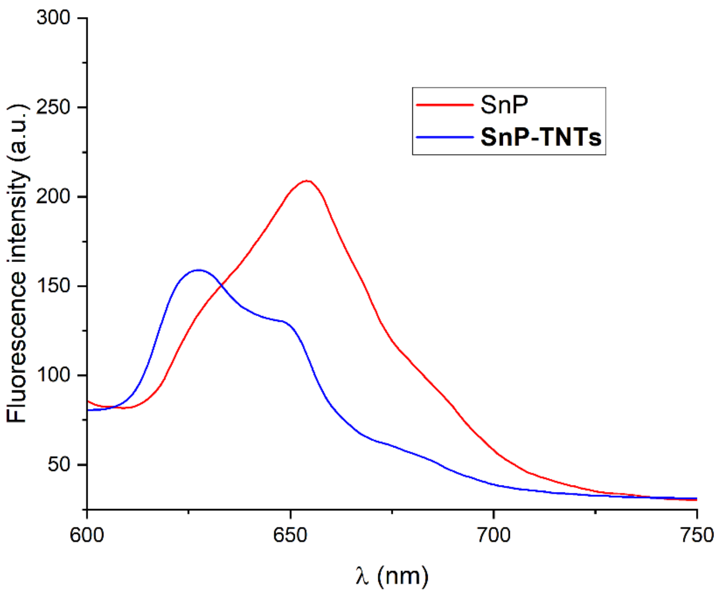

Figure S2. Fluorescence spectra of SnP and SnP-TNTs in Nujol (λ_{ex} = 550 nm).

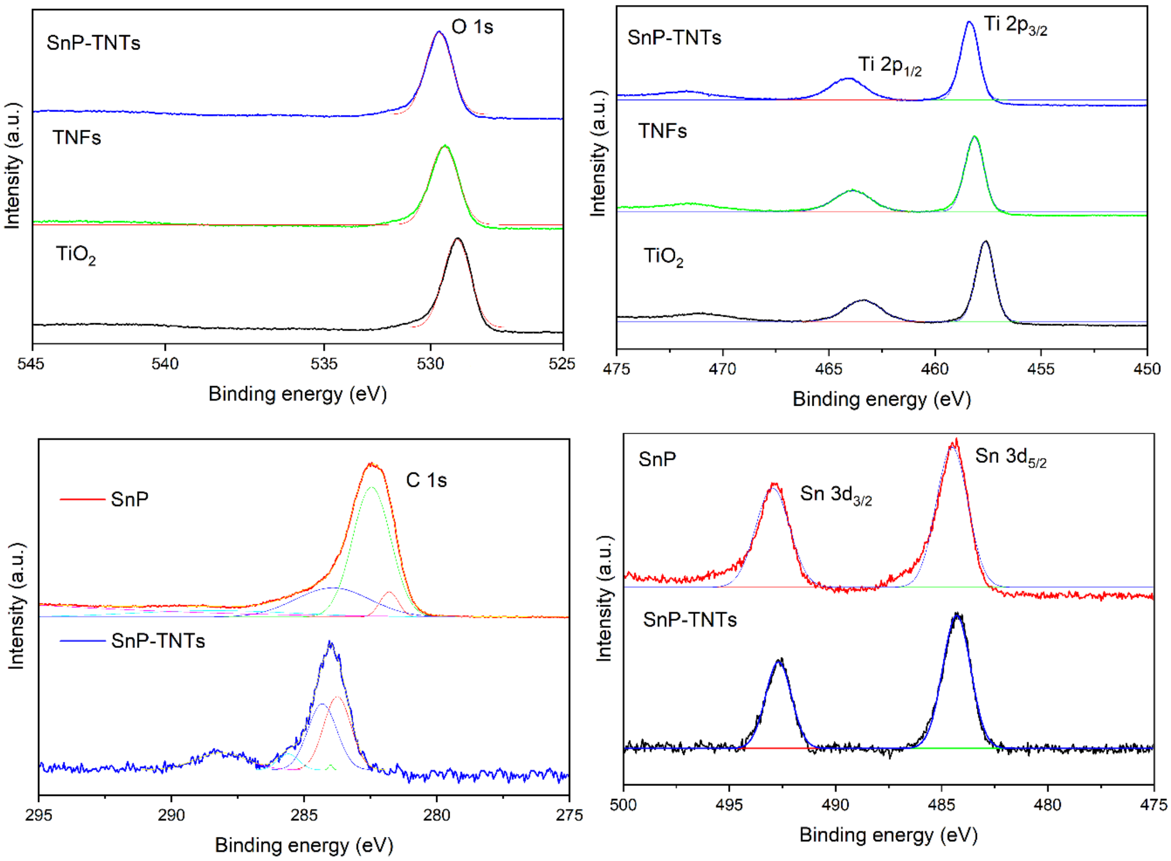

Figure S3. XPS spectra of SnP-TNTs and TiO_2 (P-25). Deconvoluted profiles of the O 1s, Ti 2p, C 1s, and Sn 3d core levels.

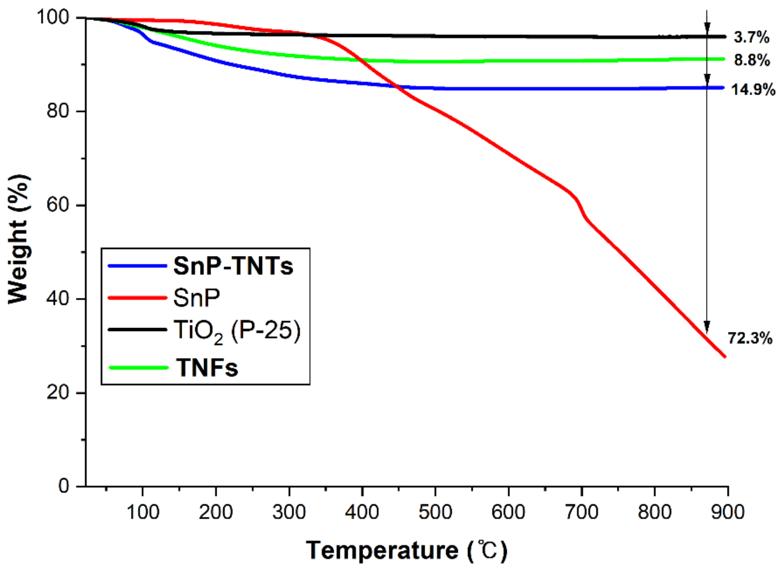

Figure S4. TGA curves of SnP, TiO_2 (P-25), TNFs, and SnP-TNTs.

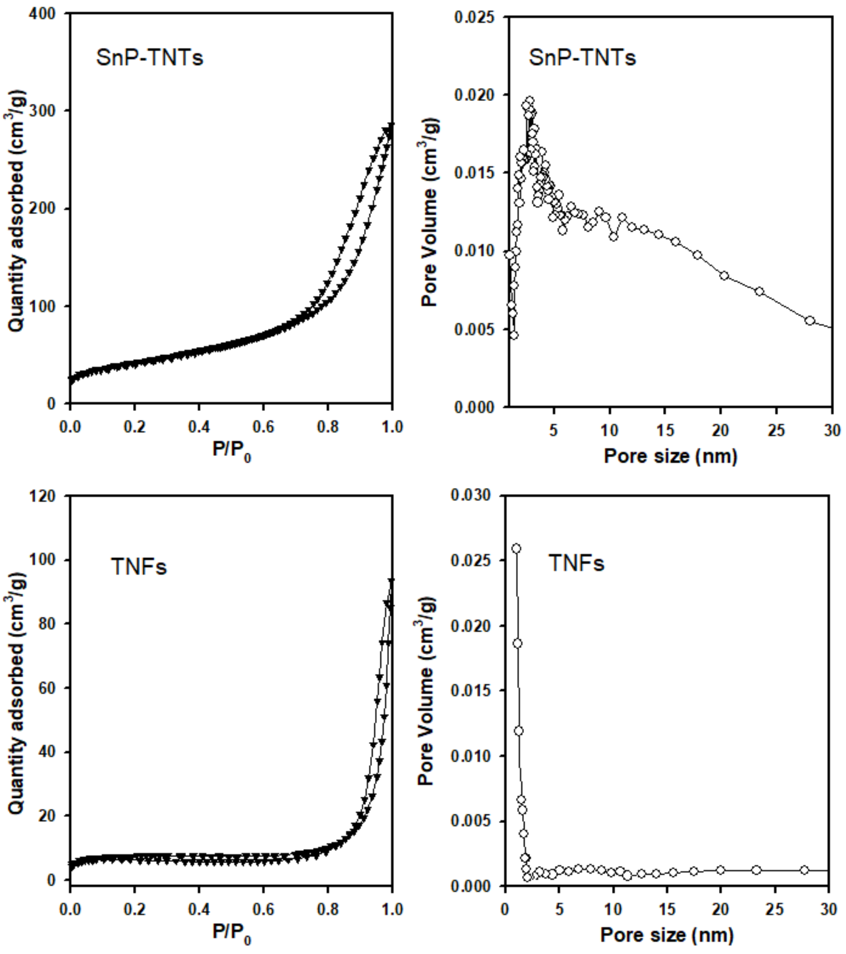

Figure S5. N_2 adsorption-desorption isotherms of SnP-TNTs and TNFs (right); corresponding pore size distribution curve (left).

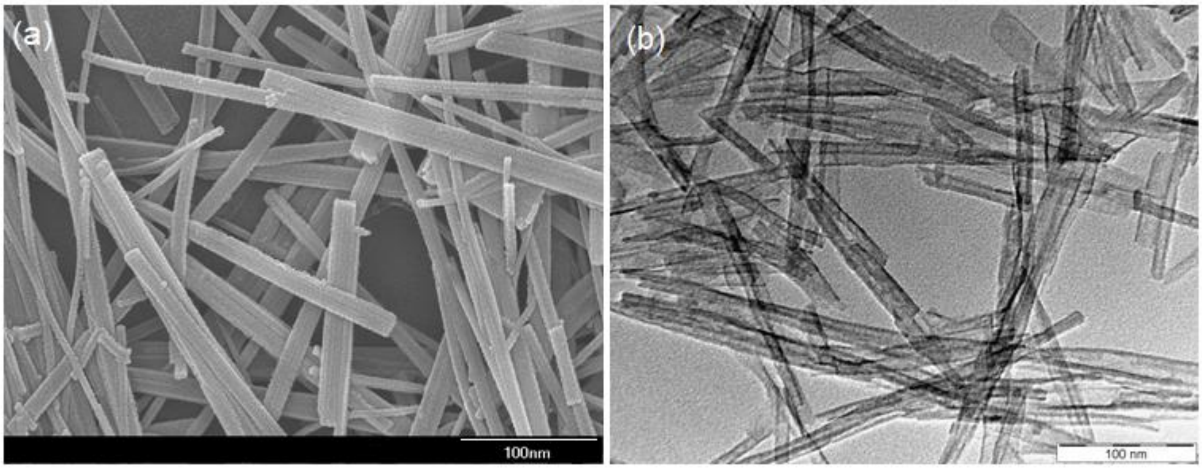

Figure S6. Enlarged FE-SEM and TEM images for SnP-TNTs.

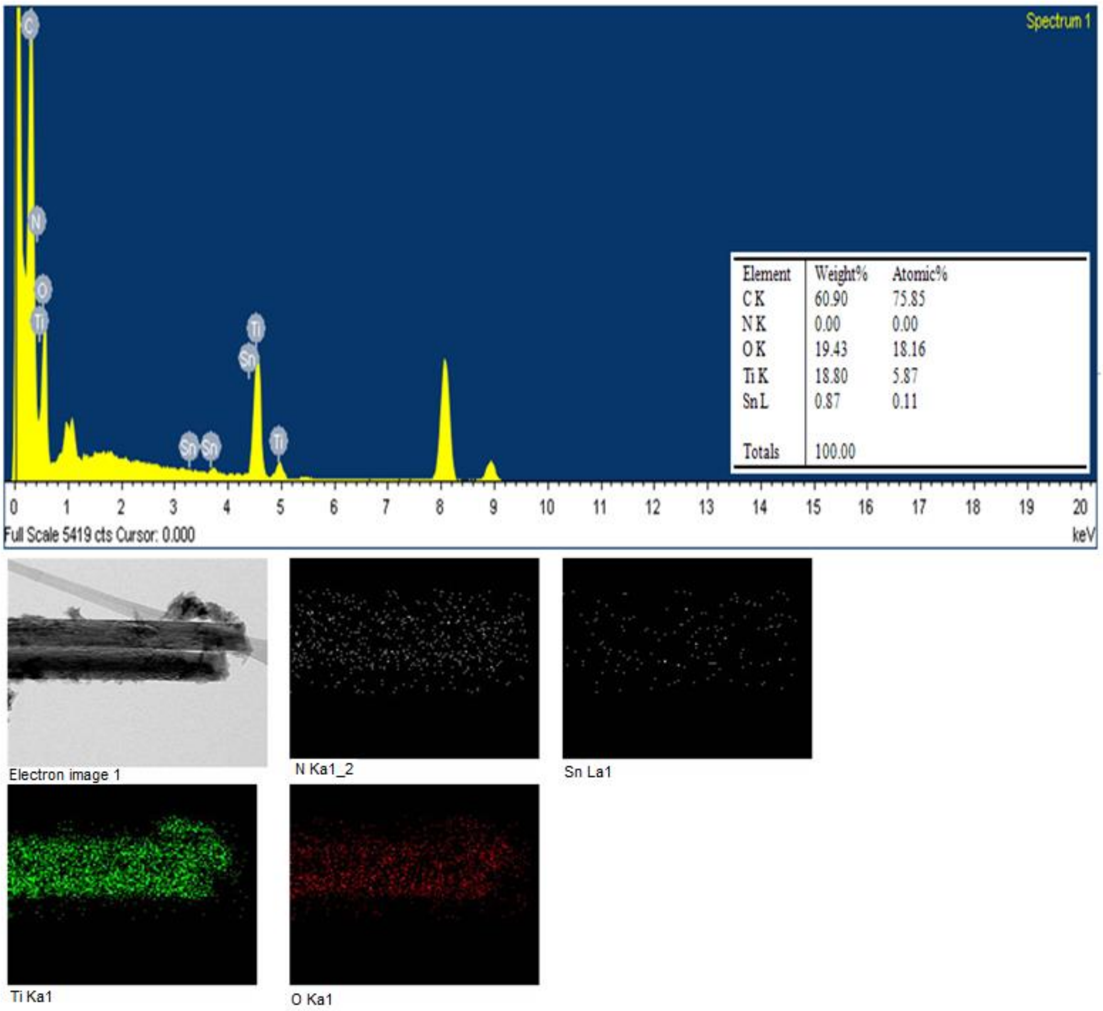

Figure S7. Energy dispersive X-ray spectroscopy (EDS) elemental maps (C, N, O, Ti, and Sn) of SnP-TNTs derived from TEM analysis.

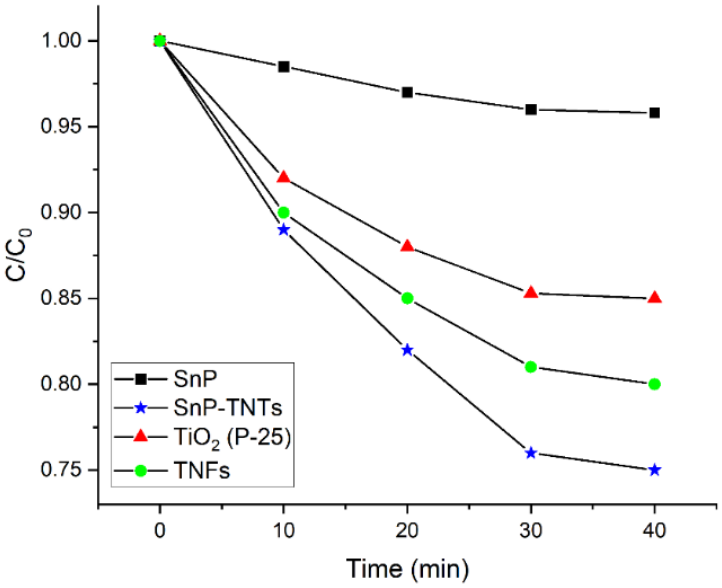

Figure S8. Time-dependent adsorption ratios for MB dye using SnP, TiO_2 (P-25), SnP-TNTs, and TNFs.

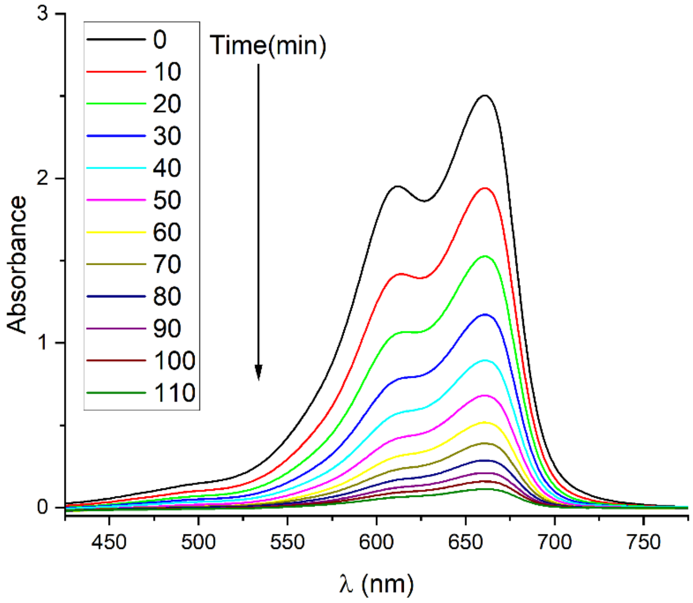

Figure S9. Visible light-driven photocatalytic degradation of MB dye in aqueous solution using SnP-TNTs.

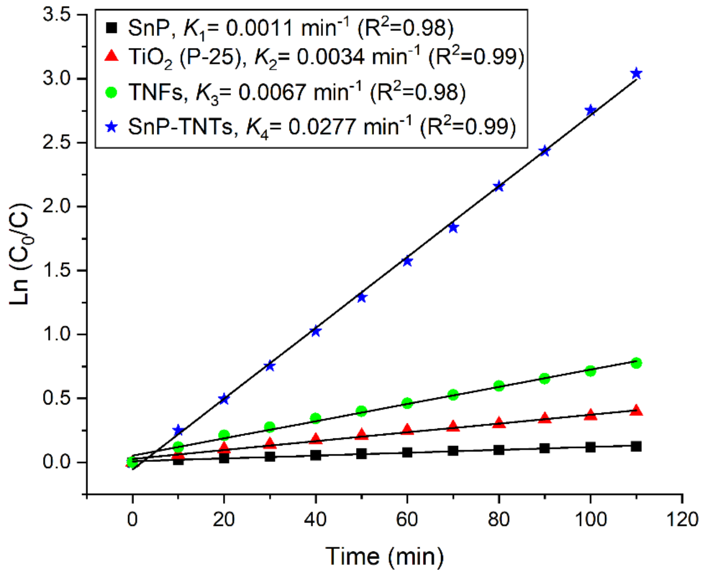

Figure S10. Comparative kinetics of photocatalytic degradation of MB dye under visible light irradiation using SnP, TiO_2 (P-25), SnP-TNTs, and TNFs as photocatalysts.

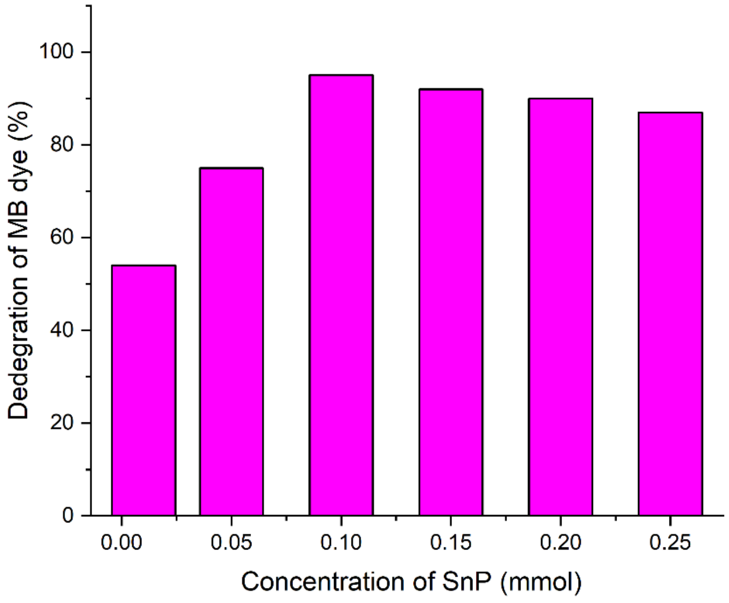

Figure S11. Comparison of MB dye degradation in the presence of different SnP-TNTs (SnP concentration relative to TiO_2 P-25).

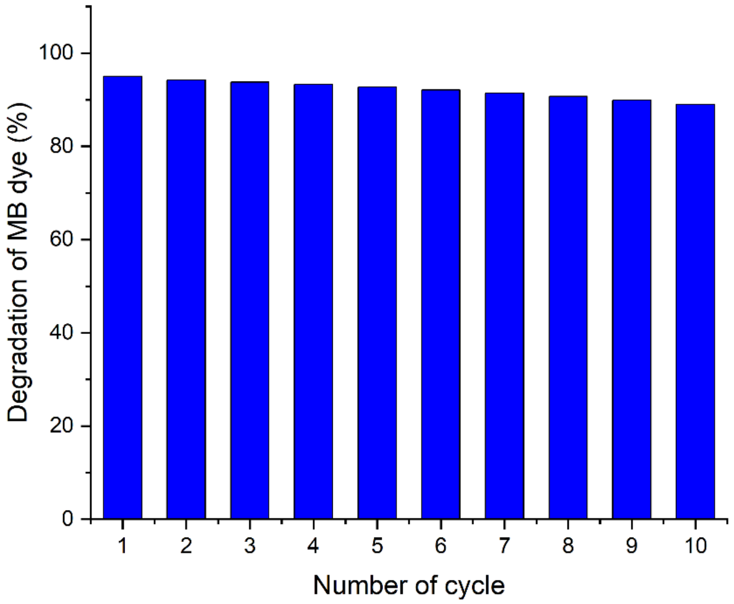

Figure S12. Cyclability of the composite photocatalyst SnP-TNTs in the degradation of MB dye.

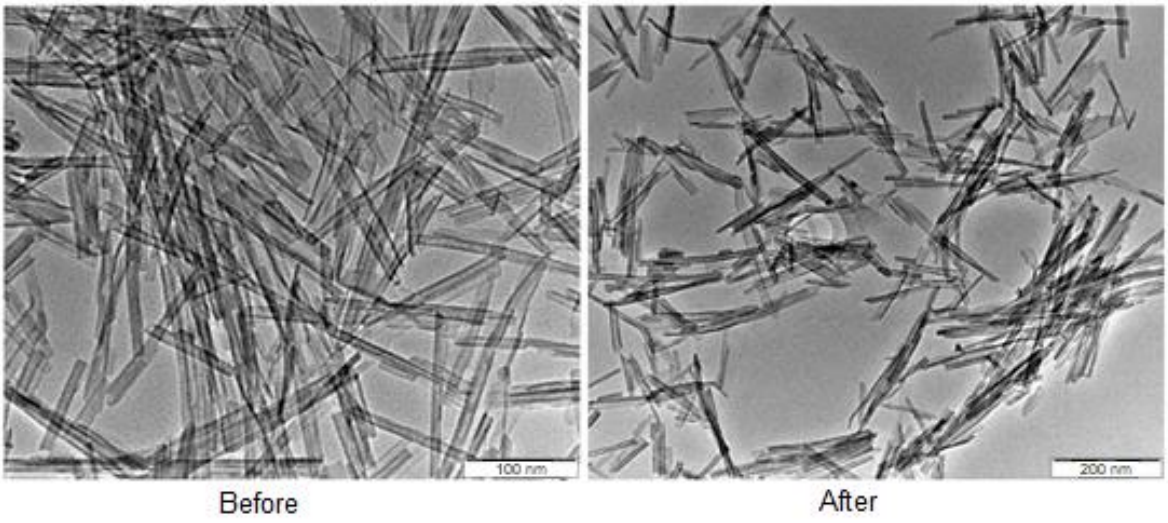

Figure S13. TEM of composite photocatalyst SnP-TNTs, before and after the MB dye degradation experiment.

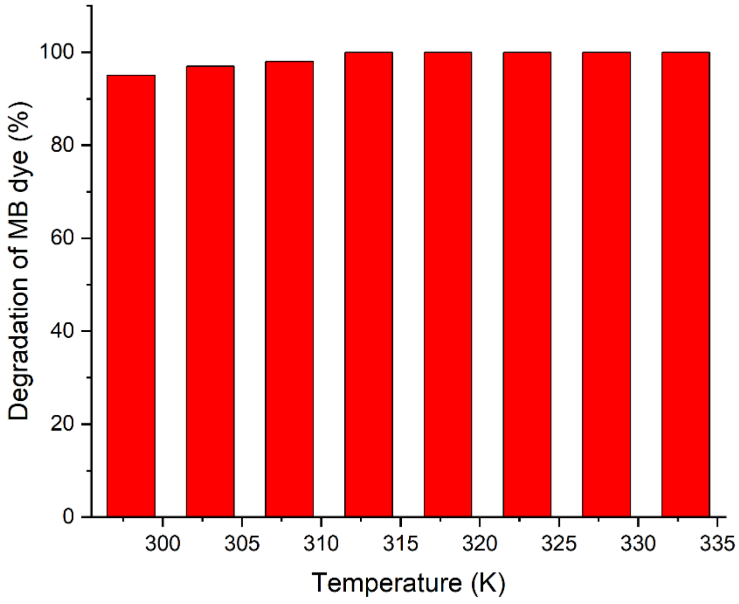

Figure S14. Temperature-dependent degradation of MB Dye in the presence of composite photocatalyst SnP-TNTs.

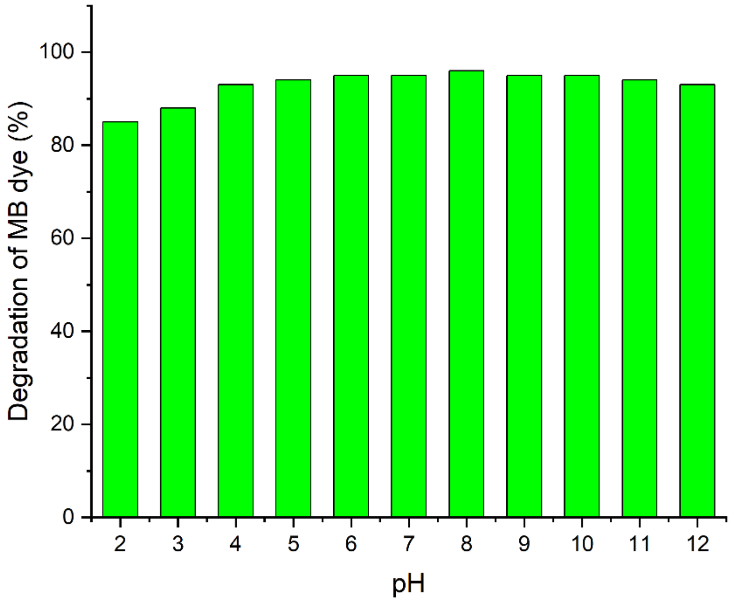

Figure S15. Effect of pH on the degradation of MB dye in the presence of the composite photocatalyst SnP-TNTs.

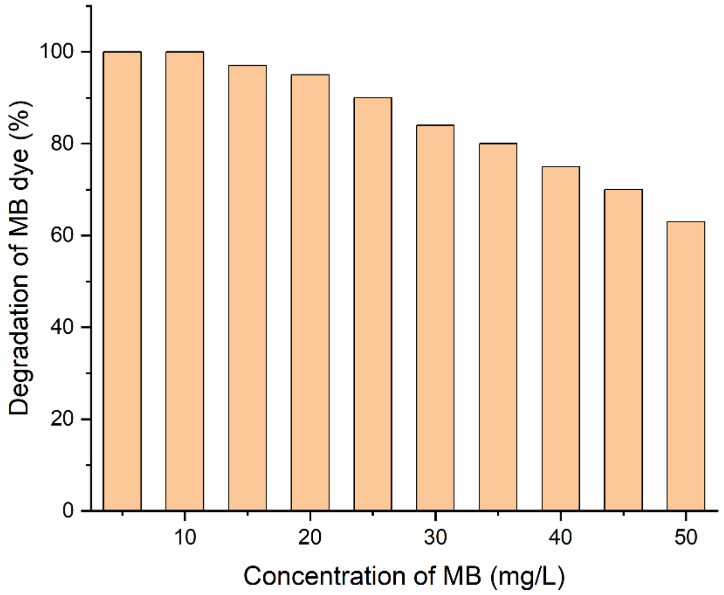

Figure S16. Effect of initial concentration of MB dye on degradation experiments using 50 mg of composite photocatalyst SnP-TNTs.

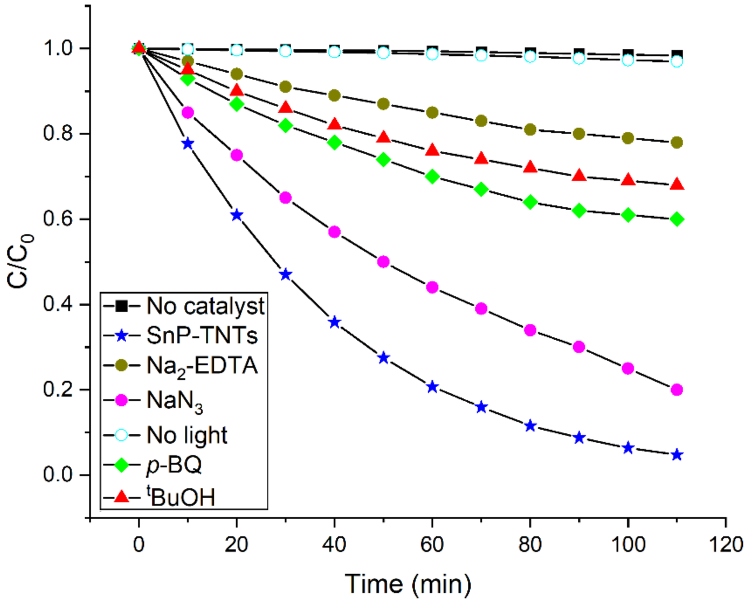

Figure S17. Photocatalytic degradation of MB dye in aqueous solution by the composite photocatalyst SnP-TNTs with different scavengers under visible light irradiation ($[Na_2\text{-}EDTA]_0 = [p\text{-}BQ]_0 = [NaN_3]_0 = [^tBuOH]_0$ = 1 mM, pH 7.0, T = 298 K).

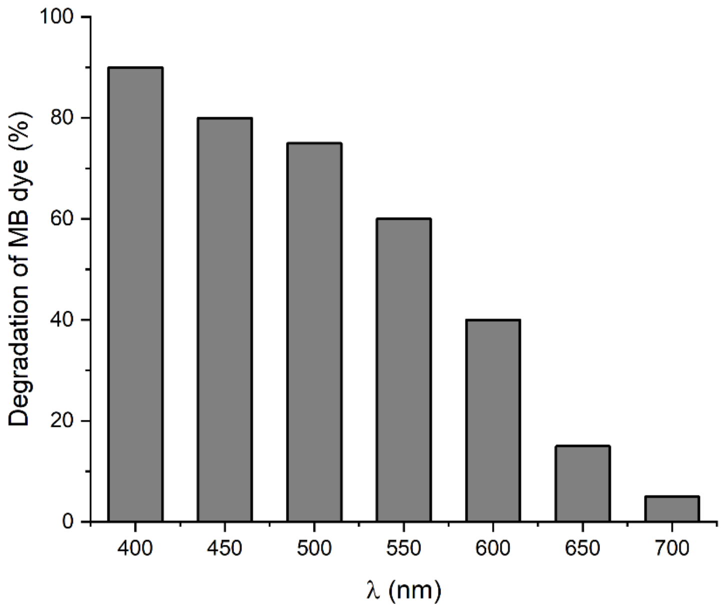

Figure S18. Photocatalytic activities of SnP-TNTs at different wavelengths for the degradation of MB dye.

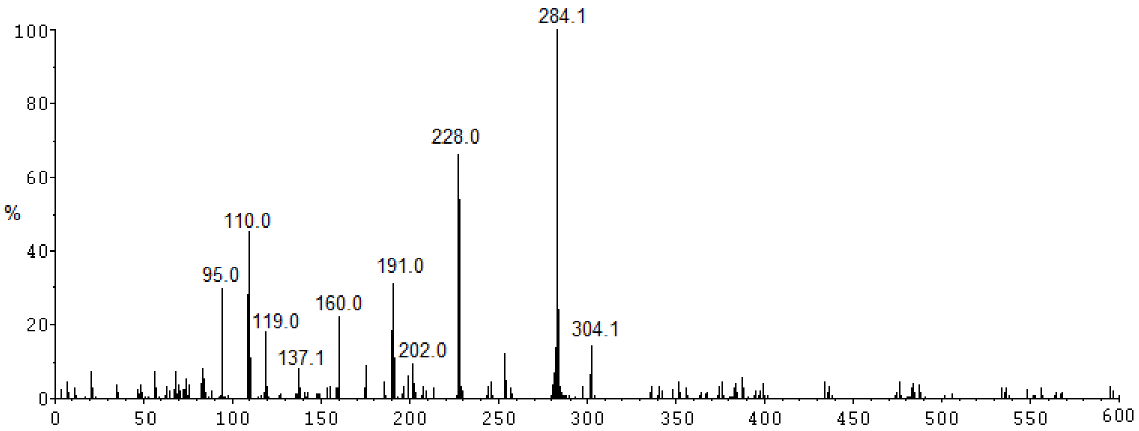

Figure S19. ESI-MS spectrum (positive ion mode) of the reaction mixture of MB dye with the composite photocatalyst SnP-TNTs after 60 min of visible light irradiation.

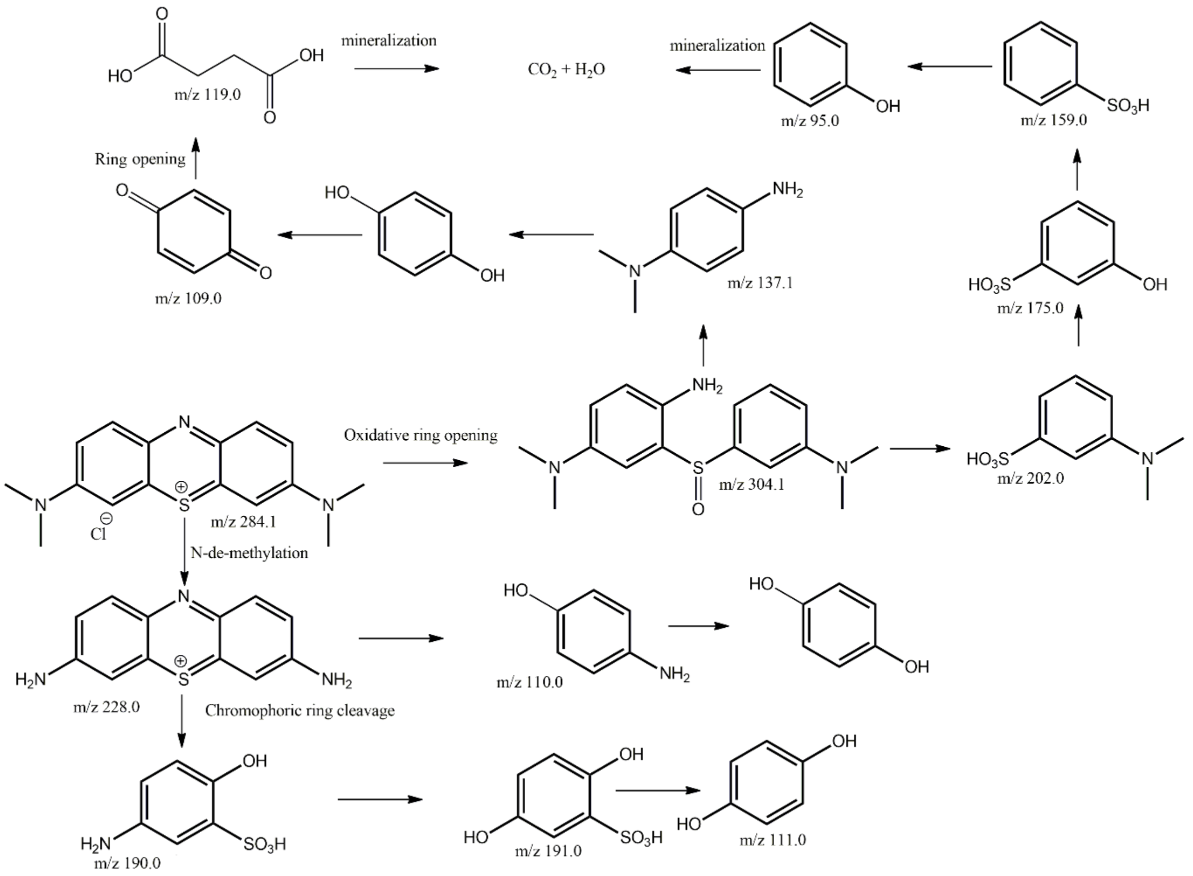

Figure S20. Possible intermediates in the degradation pathway of MB dye in the presence of composite photocatalyst SnP-TNTs.

References

1. Eskizeybek, V.; Sarı, F.; Gülce, H.; Gülce, A.; Avcı, A. Preparation of the new polyaniline/ZnO nanocomposite and its photocatalytic activity for degradation of methylene blue and malachite green dyes under uv and natural sun lights irradiations. *Appl. Catal. B* **2012**, *119*, 197−206.
2. Gülce, H.; Eskizeybek, V.; Haspulat, B.; Sarı, F.; Gülce, A.; Avcı, A. Preparation of a new polyaniline/CdO nanocomposite and investigation of its photocatalytic activity: Comparative study under UV light and natural sunlight irradiation. *Ind. Eng. Chem. Res*. **2013**, *52*, 10924−10934.
3. Kant, S.; Kalia, S.; Kumar, A. A novel nanocomposite of polyaniline and a novel nanocomposite of polyaniline and $Fe_{0.01}Ni_{0.01}Zn_{0.98}O$: Photocatalytic, electrical and antibacterial properties. *J. Alloy. Compd*. **2013**, *578*, 249−256.
4. Chen, W.; Xiao, H.; Xu, H.; Ding, T.; Gu, Y. Photodegradation of Methylene Blue by TiO_2-Fe_3O_4-Bentonite Magnetic Nanocomposite. *Int. J. Photoenergy* **2015**, *2015*, 591428.
5. Tian, H.; Wan, C.; Xue, X.; Hu, X.; Wang, X. Effective Electron Transfer Pathway of the Ternary TiO_2/RGO/Ag Nanocomposite with Enhanced Photocatalytic Activity under Visible Light. *Catalysts* **2017**, *7*, 156.
6. Singh, J.; Chang, Y.-Y.; Koduru, J.R.; Yang, J.-K. Potential Degradation of Methylene Blue (MB) by Nano-Metallic Particles: A Kinetic Study and Possible Mechanism of MB Degradation. *Environ. Eng. Res.* **2017**, *23*, 1−9.
6. Yang, C.; Dong, W.; Cui, G.; Zhao, Y.; Shi, X.; Xia, X.; Tang, B.; Wang, W. Highly Efficient Photocatalytic Degradation of Methylene Blue by P2ABSA-Modified TiO_2 Nanocomposite Due to the Photosensitization Synergetic Effect of TiO_2 and P2ABSA. *RSC Adv.* **2017**, *7*, 23699−23708.
8. Yang, C.; Zhang, M.; Dong, W.; Cui, G.; Ren, Z.; Wang, W. Highly efficient photocatalytic degradation of methylene blue by PoPD/TiO_2 nanocomposite. *PLoS ONE* **2017**, *12*, e0174104.
9. Ahmad, J.; Sofi, F. A.; Mehraj, O.; Majid, K. Fabrication of highly photocatalytic active anatase TiO_2-graphene oxide heterostructures via solid phase ball milling for environmental remediation. *Surf. Interfaces* **2018**, *13*, 186−195.

10. Azeez, F.; Al-Hetlani, E.; Arafa, M.; Abdelmonem, Y.; Abdel Nazeer, A.; Amin, M.; Madkour, M. The effect of surface charge on photocatalytic degradation of methylene blue dye using chargeable titania nanoparticles. *Sci. Rep.* **2018**, *8*, 1−9.
11. Adeleke, J.T.; Theivasanthi, T.; Thiruppathi, M.; Swaminathan, M.; Akomolafe, T.; Alabi, A.B. Photocatalytic degradation of methylene blue by ZnO/NiFe_2O_4 nanoparticles. *Appl. Surf. Sci.* **2018**, *455*, 195−200.
12. Rajendran, R.; Varadharajan, K.; Jayaraman, V.; Singaram, B.; Jeyaram, J. Photocatalytic degradation of metronidazole and methylene blue by PVA-assisted Bi_2WO_6–CdS nanocomposite film under visible light irradiation. *Appl. Nanosci.* **2018**, *8,* 61−78.
13. Benzaouak, A.; Ellouzi, I.; Ouanji, F.; Touach, N.; Kacimi, M.; Ziyad, M.; Mahi, M.El.; Lofti, E.M. Photocatalytic degradation of methylene blue (MB) dye in aqueous solution by ferroelectric $Li_{1-x}Ta_{1-x}W_xO_3$ materials. *Colloids Surfaces A Physicochem. Eng. Asp*., **2018**, *553*, 586−592.
14. Nuengmatcha, P.; Porrawatkul, P.; Chanthai, S.; Sricharoen, P.; Limchoowong, N. Enhanced photocatalytic degradation of methylene blue using Fe_2O_3/graphene/CuO nanocomposites under visible light. *J. Environ. Chem. Eng.* **2019**, *7,* 103438.
15. Zhang, D.; Dai, F.; Zhang, P.; Ana, Z.; Zhao, Y.; Chen, L. The photodegradation of methylene blue in water with PVDF/GO/ZnO composite membrane. *Mater. Sci. Eng. C* **2019**, *96*, 684−692.
16. Motola, M.; Baudys, M.; Zazpe, R.; Krbal, M.; Michalička, J.; Rodriguez-Pereira, J.; Pavliňák, D.; Přikryl, J.; Hromádko, L.; Sopha, H.; Krysa, J.; Macak, J.M. 2D MoS_2 Nanosheets on 1D Anodic TiO_2 Nanotube Layers: An Efficient Co-Catalyst for Liquid and Gas Phase Photocatalysis. *Nanoscale* **2019**, *11*, 23126−23131.
17. Majumder, D.; Chakraborty, I.; Mandal, K.; Roy, S. Facet-Dependent Photodegradation of Methylene Blue Using Pristine CeO_2 Nanostructures. *ACS Omega* **2019**, *4,* 4243−4251.
18. Singh, N.; Jana, S.; Singh, G.P.; Dey, R.K. Graphene-supported TiO_2: Study of promotion of charge carrier in photocatalytic water splitting and methylene blue dye degradation. *Adv. Compos. Hybrid. Mater* **2020**, *3*, 127−140.
19. Vasiljevic, Z.Z.; Dojcinovic, M.P.; Vujancevic, J.D.; Jankovic-Castvan, I.; Ognjanovic, M.; Tadic, N.B.; Stojadinovic, S.; Brankovic, G.O.; Nikolic, M.V. Photocatalytic

degradation of methylene blue under natural sunlight using iron titanate nanoparticles prepared by a modified sol–gel method, *R. Soc. Open Sci.*, **2020**, *7* , 200708.

20. Alkaykh, S.; Mbarek, A.; Ali-Shattle, E.E. Photocatalytic degradation of methylene blue dye in aqueous solution by $MnTiO_3$ nanoparticles under sunlight irradiation. *Heliyon* **2020**, *6*, e03663.
21. Rahman, Q.I.; Hasan, H.; Ali, A.; Mehta, S.K.; Raja, M.A.; Ahmad, N.; Khan, A.R.; Muddassir, M. Synthesis and Characterizations of Nitrogen (N) Doped Strontium Titanate ($SrTiO_3$) Nanoparticles for Enhanced Visible Light Driven Photocatalytic Degradation. *J. Nanosci. Nanotechnol.* **2020**, *20*, 6475−6481.
22. Makeswari, M.; Saraswathi, P. Photo catalytic degradation of methylene blue and methyl orange from aqueous solution using solar light onto chitosan bi-metal oxide composite. *SN Appl. Sci.* **2020**, *2*, 336.
23. Sarkar, D.; Ganguli, S.; Praveen, A.E.; Mahalingam, V. Defect induced "super mop" like behavior of Eu^{3+}-doped hierarchical Bi_2SiO_5 nanoparticles for improved catalytic and adsorptive behaviour. *Mater. Adv.*, **2020**, *1*, 2019−2032.
24. Ahmed, M.K.; El-Naggar, M.E.; Aldalbahi, A.; El-Newehy, M.H.; Menazea, A.A. Methylene Blue Degradation under Visible Light of Metallic Nanoparticles Scattered into Graphene Oxide Using Laser Ablation Technique in Aqueous Solutions. *J. Mol. Liq.* **2020**, *315*, 113794.
25. Ghoniem, M.G.; Talab, S.A.; Modwi, A.K.; Taha, K.K. Exploration of Methylene Blue Degradation over ZnO Nanorods Mechanism using Scavenging Reagents. *Orient J. Chem*., **2021**, 37, 609−618.
26. Abd El Khalk, A. A.; Betiha, M. A.; Mansour, A. S.; Abd El Wahed, M. G.; Al-Sabagh, A. M. High Degradation of Methylene Blue Using a New Nanocomposite Based on Zeolitic Imidazolate Framework-8. *ACS Omega* **2021**, *6*, 26210−26220.
27. Shokoohi, R.; Khazaei, M.; Godini, K.; Azarian, G.; Latifi, Z.; Javadimanesh, L.; Zolghadr Nasab, H. Degradation and Mineralization of Methylene Blue Dye by Peroxymonosulfate/Mn_3O_4 Nanoparticles Using Central Composite Design: Kinetic Study. *Inorg. Chem. Commun.* **2021**, *127*, 108501.
28. Arias, M.-C.; Aguilar, C.; Piza, M.; Zarazua, E.; Anguebes, F.; Anguebes, F.; Anguebes, F.; Anguebes, F.; Cordova, V. Removal of the Methylene Blue Dye (MB) with Catalysts of Au-TiO_2: Kinetic and Degradation Pathway. *Mod. Res. Catal.* **2021**, *10*, 1−14.

29. Duan, K.; Que, T.; Koppala, S.; Balan, R.; Lokesh, B.; Pillai, R.; David, S.; Karthikeyan, P.; Ramamoorthy, S.; Lekshmi, I. A facile route to synthesize n-SnO_2/p-$CuFe_2O_4$ to rapidly degrade toxic methylene blue dye under natural sunlight. *RSC Adv.*, **2022**, *12*, 16544−16553.
30. Mishra, S.; Chakinala, N.; Chakinala, A.G.; Surolia, P.K. Photocatalytic degradation of methylene blue using monometallic and bimetallic Bi-Fe doped TiO_2. *Catal. Commun.*, **2022**, *171*, 106518.
31. BinSabt, M.; Sagar, V.; Singh, J.; Rawat, M.; Shaban, M. Green Synthesis of CS-TiO_2 NPs for Efficient Photocatalytic Degradation of Methylene Blue Dye. *Polymers* **2022**, *14*, 2677.
32. Quiton, K.G.N.; Lu, M.-C.; Huang, Y.-H. Synergistic degradation of methylene blue by novel Fe-Co bimetallic catalyst supported on waste silica in photo-Fenton-like system. *Sustain. Environ. Res.*, **2022**, *32*, 21.
33. Abdullah, M.; John, P.; Ashiq, M.N.; Manzoor, S.; Ghori, M. I.; Nisa, M.U.; Abid, A.G.; Butt, K.Y.; Ahmed, S. Development of CuO/CuS/MnO_2 ternary nanocomposite for visible light-induced photocatalytic degradation of methylene blue, *Nanotechnol. Environ. Eng*. **2022**, *1*, 1−11.
34. Shee, N.K.; Kim, M.K.; Kim, H.-J. Supramolecular Porphyrin Nanostructures Based on Coordination-Driven Self-Assembly and Their Visible Light Catalytic Degradation of Methylene Blue Dye. *Nanomaterials* **2020**, *10*, 2314.
35. Shee, N.K.; Kim, H.-J. Coordination framework materials fabricated by the self-assembly of Sn(IV) porphyrins with Ag(I) ions for the photocatalytic degradation of organic dyes in wastewater. *Inorg. Chem. Front.* **2022**, *9*, 1270–1280.
